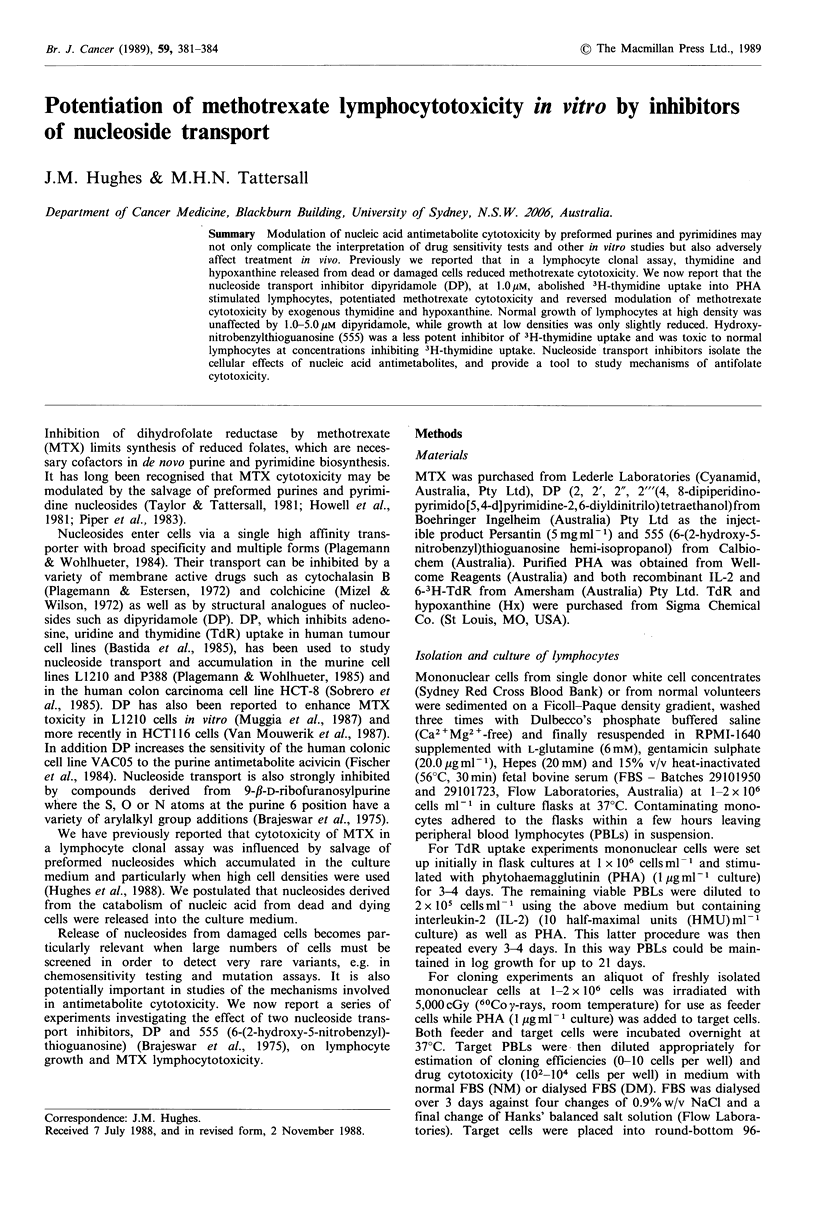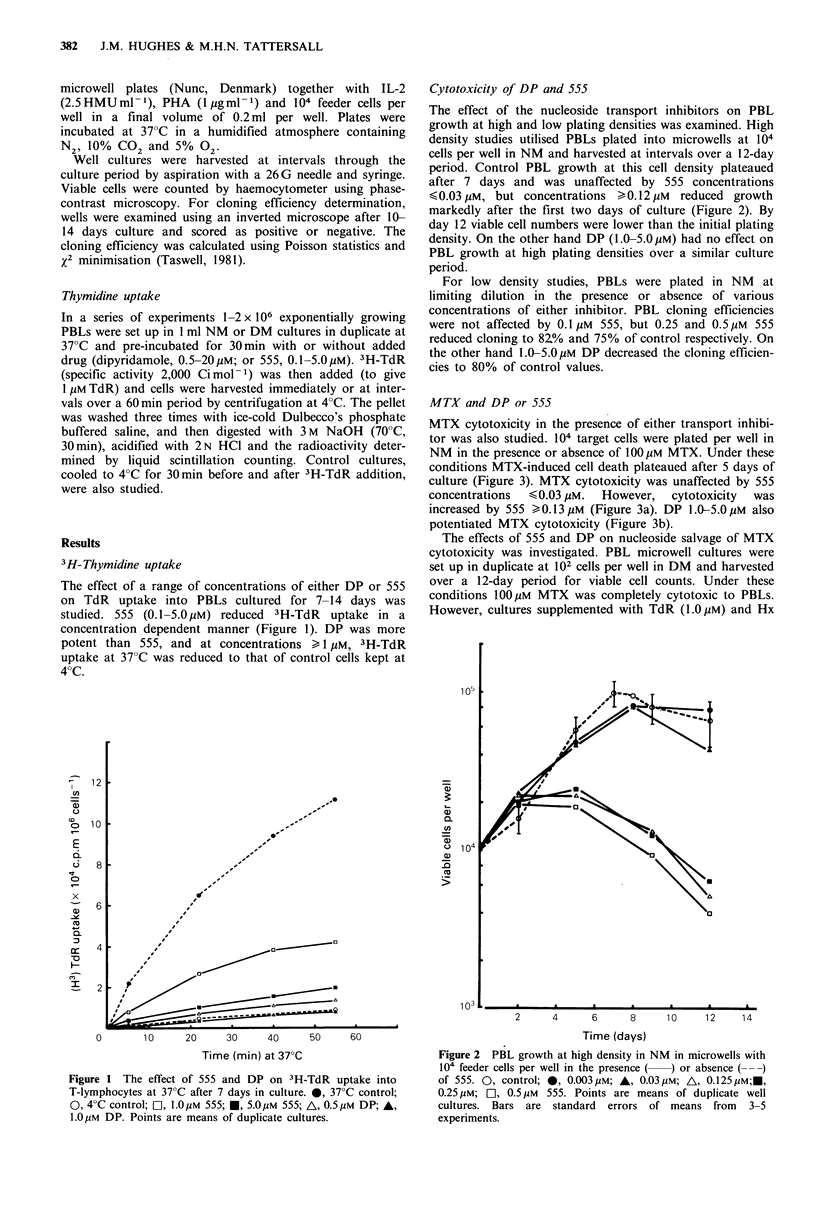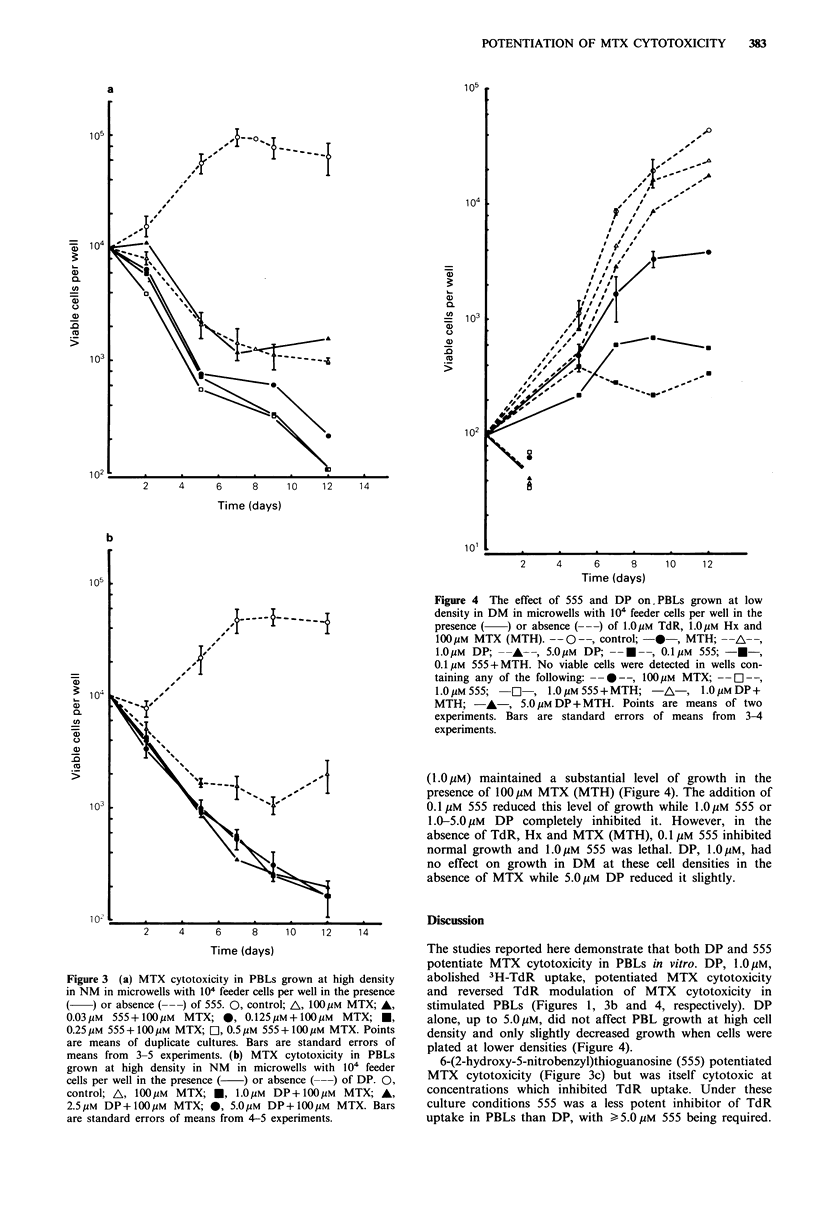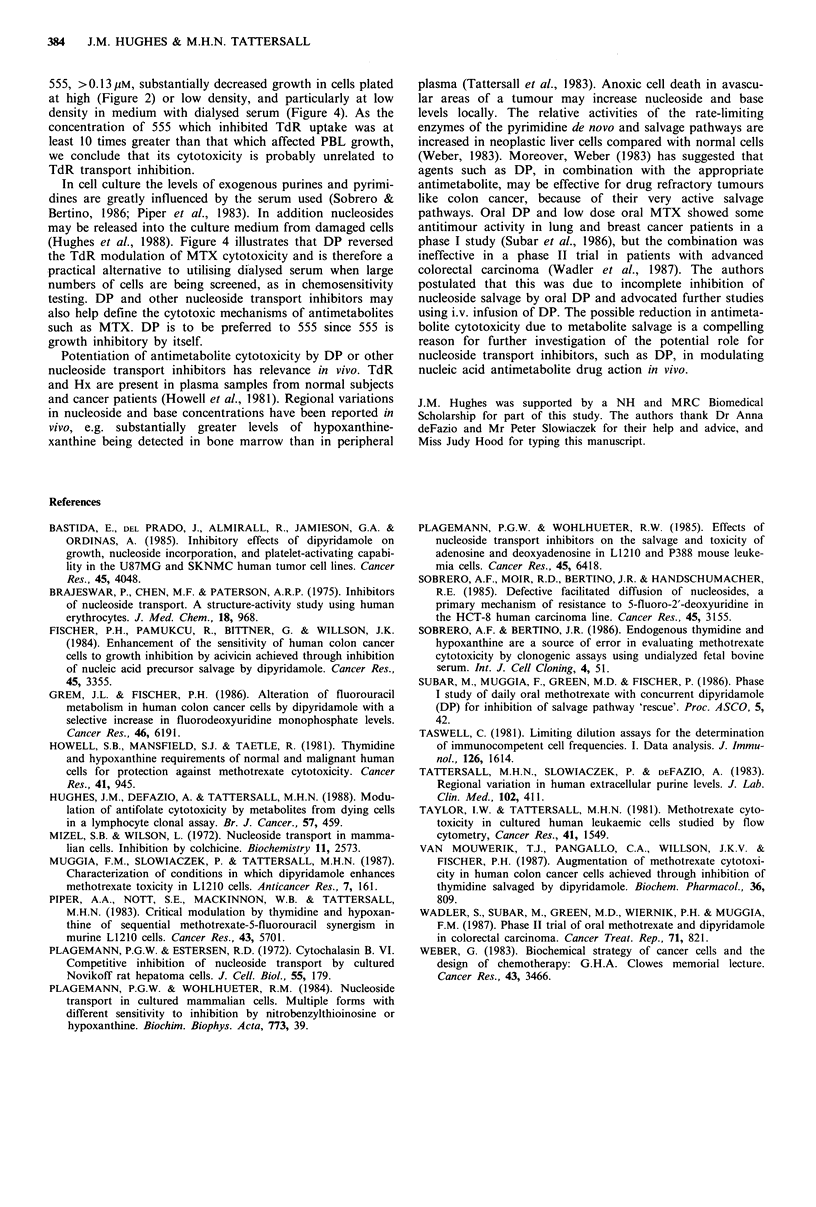# Potentiation of methotrexate lymphocytotoxicity in vitro by inhibitors of nucleoside transport.

**DOI:** 10.1038/bjc.1989.76

**Published:** 1989-03

**Authors:** J. M. Hughes, M. H. Tattersall

**Affiliations:** Department of Cancer Medicine, University of Sydney, N.S.W., Australia.

## Abstract

Modulation of nucleic acid antimetabolite cytotoxicity by preformed purines and pyrimidines may not only complicate the interpretation of drug sensitivity tests and other in vitro studies but also adversely affect treatment in vivo. Previously we reported that in a lymphocyte clonal assay, thymidine and hypoxanthine released from dead or damaged cells reduced methotrexate cytotoxicity. We now report that the nucleoside transport inhibitor dipyridamole (DP), at 1.0 microM, abolished 3H-thymidine uptake into PHA stimulated lymphocytes, potentiated methotrexate cytotoxicity and reversed modulation of methotrexate cytotoxicity by exogenous thymidine and hypoxanthine. Normal growth of lymphocytes at high density was unaffected by 1.0-5.0 microM dipyridamole, while growth at low densities was only slightly reduced. Hydroxy-nitrobenzylthioguanosine (555) was a less potent inhibitor of 3H-thymidine uptake and was toxic to normal lymphocytes at concentrations inhibiting 3H-thymidine uptake. Nucleoside transport inhibitors isolate the cellular effects of nucleic acid antimetabolites, and provide a tool to study mechanisms of antifolate cytotoxicity.


*Br. J. Cancer* (1989), **59**, 381–384 © The Macmillan Press Ltd., 1989

### Potentiation of methotrexate lymphocytotoxicity *in vitro* by inhibitors of nucleoside transport

J.M. Hughes & M.H.N. Tattersall

*Department of Cancer Medicine, Blackburn Building, University of Sydney, N.S.W. 2006, Australia.*

**Summary** Modulation of nucleic acid antimetabolite cytotoxicity by preformed purines and pyrimidines may not only complicate the interpretation of drug sensitivity tests and other *in vitro* studies but also adversely affect treatment *in vivo*. Previously we reported that in a lymphocyte clonal assay, thymidine and hypoxanthine released from dead or damaged cells reduced methotrexate cytotoxicity. We now report that the nucleoside transport inhibitor dipyridamole (DP), at 1.0 $\mu$M, abolished $^3$H-thymidine uptake into PHA stimulated lymphocytes, potentiated methotrexate cytotoxicity and reversed modulation of methotrexate cytotoxicity by exogenous thymidine and hypoxanthine. Normal growth of lymphocytes at high density was unaffected by 1.0–5.0 $\mu$M dipyridamole, while growth at low densities was only slightly reduced. Hydroxynitrobenzylthioguanosine (555) was a less potent inhibitor of $^3$H-thymidine uptake and was toxic to normal lymphocytes at concentrations inhibiting $^3$H-thymidine uptake. Nucleoside transport inhibitors isolate the cellular effects of nucleic acid antimetabolites, and provide a tool to study mechanisms of antifolate cytotoxicity.

Inhibition of dihydrofolate reductase by methotrexate (MTX) limits synthesis of reduced folates, which are necessary cofactors in *de novo* purine and pyrimidine biosynthesis. It has long been recognised that MTX cytotoxicity may be modulated by the salvage of preformed purines and pyrimidine nucleosides (Taylor & Tattersall, 1981; Howell *et al.*, 1981; Piper *et al.*, 1983).

Nucleosides enter cells via a single high affinity transporter with broad specificity and multiple forms (Plagemann & Wohlhueter, 1984). Their transport can be inhibited by a variety of membrane active drugs such as cytochalasin B (Plagemann & Estersen, 1972) and colchicine (Mizel & Wilson, 1972) as well as by structural analogues of nucleosides such as dipyridamole (DP). DP, which inhibits adenosine, uridine and thymidine (TdR) uptake in human tumour cell lines (Bastida *et al.*, 1985), has been used to study nucleoside transport and accumulation in the murine cell lines L1210 and P388 (Plagemann & Wohlhueter, 1985) and in the human colon carcinoma cell line HCT-8 (Sobrero *et al.*, 1985). DP has also been reported to enhance MTX toxicity in L1210 cells *in vitro* (Muggia *et al.*, 1987) and more recently in HCT116 cells (Van Mouwerik *et al.*, 1987). In addition DP increases the sensitivity of the human colonic cell line VAC05 to the purine antimetabolite acivicin (Fischer *et al.*, 1984). Nucleoside transport is also strongly inhibited by compounds derived from 9-$\beta$-D-ribofuranosylpurine where the S, O or N atoms at the purine 6 position have a variety of arylalkyl group additions (Brajeswar *et al.*, 1975).

We have previously reported that cytotoxicity of MTX in a lymphocyte clonal assay was influenced by salvage of preformed nucleosides which accumulated in the culture medium and particularly when high cell densities were used (Hughes *et al.*, 1988). We postulated that nucleosides derived from the catabolism of nucleic acid from dead and dying cells were released into the culture medium.

Release of nucleosides from damaged cells becomes particularly relevant when large numbers of cells must be screened in order to detect very rare variants, e.g. in chemosensitivity testing and mutation assays. It is also potentially important in studies of the mechanisms involved in antimetabolite cytotoxicity. We now report a series of experiments investigating the effect of two nucleoside transport inhibitors, DP and 555 (6-(2-hydroxy-5-nitrobenzyl)-thioguanosine) (Brajeswar *et al.*, 1975), on lymphocyte growth and MTX lymphocytotoxicity.

Correspondence: J.M. Hughes.
Received 7 July 1988, and in revised form, 2 November 1988.

### Methods

### *Materials*

MTX was purchased from Lederle Laboratories (Cyanamid, Australia, Pty Ltd), DP (2, 2′, 2″, 2‴(4, 8-dipiperidino-pyrimido[5,4-d]pyrimidine-2,6-diyldinitrilo)tetraethanol) from Boehringer Ingelheim (Australia) Pty Ltd as the injectible product Persantin (5 mg ml$^{-1}$) and 555 (6-(2-hydroxy-5-nitrobenzyl)thioguanosine hemi-isopropanol) from Calbiochem (Australia). Purified PHA was obtained from Wellcome Reagents (Australia) and both recombinant IL-2 and 6-$^3$H-TdR from Amersham (Australia) Pty Ltd. TdR and hypoxanthine (Hx) were purchased from Sigma Chemical Co. (St Louis, MO, USA).

### *Isolation and culture of lymphocytes*

Mononuclear cells from single donor white cell concentrates (Sydney Red Cross Blood Bank) or from normal volunteers were sedimented on a Ficoll–Paque density gradient, washed three times with Dulbecco's phosphate buffered saline ($Ca^{2+}Mg^{2+}$-free) and finally resuspended in RPMI-1640 supplemented with L-glutamine (6 mM), gentamicin sulphate (20.0 $\mu$g ml$^{-1}$), Hepes (20 mM) and 15% v/v heat-inactivated (56°C, 30 min) fetal bovine serum (FBS – Batches 29101950 and 29101723, Flow Laboratories, Australia) at $1–2 \times 10^6$ cells ml$^{-1}$ in culture flasks at 37°C. Contaminating monocytes adhered to the flasks within a few hours leaving peripheral blood lymphocytes (PBLs) in suspension.

For TdR uptake experiments mononuclear cells were set up initially in flask cultures at $1 \times 10^6$ cells ml$^{-1}$ and stimulated with phytohaemagglutinin (PHA) (1 $\mu$g ml$^{-1}$ culture) for 3–4 days. The remaining viable PBLs were diluted to $2 \times 10^5$ cells ml$^{-1}$ using the above medium but containing interleukin-2 (IL-2) (10 half-maximal units (HMU) ml$^{-1}$ culture) as well as PHA. This latter procedure was then repeated every 3–4 days. In this way PBLs could be maintained in log growth for up to 21 days.

For cloning experiments an aliquot of freshly isolated mononuclear cells at $1–2 \times 10^6$ cells was irradiated with 5,000 cGy ($^{60}$Co $\gamma$-rays, room temperature) for use as feeder cells while PHA (1 $\mu$g ml$^{-1}$ culture) was added to target cells. Both feeder and target cells were incubated overnight at 37°C. Target PBLs were then diluted appropriately for estimation of cloning efficiencies (0–10 cells per well) and drug cytotoxicity ($10^2$–$10^4$ cells per well) in medium with normal FBS (NM) or dialysed FBS (DM). FBS was dialysed over 3 days against four changes of 0.9% w/v NaCl and a final change of Hanks' balanced salt solution (Flow Laboratories). Target cells were placed into round-bottom 96-

microwell plates (Nunc, Denmark) together with IL-2 (2.5 HMU $ml^{-1}$), PHA (1 $\mu g ml^{-1}$) and $10^4$ feeder cells per well in a final volume of 0.2 ml per well. Plates were incubated at 37°C in a humidified atmosphere containing $N_2$, 10% $CO_2$ and 5% $O_2$.

Well cultures were harvested at intervals through the culture period by aspiration with a 26 G needle and syringe. Viable cells were counted by haemocytometer using phase-contrast microscopy. For cloning efficiency determination, wells were examined using an inverted microscope after 10–14 days culture and scored as positive or negative. The cloning efficiency was calculated using Poisson statistics and $\chi^2$ minimisation (Taswell, 1981).

*Thymidine uptake*

In a series of experiments $1\text{–}2 \times 10^6$ exponentially growing PBLs were set up in 1 ml NM or DM cultures in duplicate at 37°C and pre-incubated for 30 min with or without added drug (dipyridamole, 0.5–20 $\mu$M; or 555, 0.1–5.0 $\mu$M). $^3$H-TdR (specific activity 2,000 Ci $mol^{-1}$) was then added (to give 1 $\mu$M TdR) and cells were harvested immediately or at intervals over a 60 min period by centrifugation at 4°C. The pellet was washed three times with ice-cold Dulbecco's phosphate buffered saline, and then digested with 3 M NaOH (70°C, 30 min), acidified with 2 N HCl and the radioactivity determined by liquid scintillation counting. Control cultures, cooled to 4°C for 30 min before and after $^3$H-TdR addition, were also studied.

### Results

*$^3$H-Thymidine uptake*

The effect of a range of concentrations of either DP or 555 on TdR uptake into PBLs cultured for 7–14 days was studied. 555 (0.1–5.0 $\mu$M) reduced $^3$H-TdR uptake in a concentration dependent manner (Figure 1). DP was more potent than 555, and at concentrations $\geq 1$ $\mu$M, $^3$H-TdR uptake at 37°C was reduced to that of control cells kept at 4°C.

**Figure 1** The effect of 555 and DP on $^3$H-TdR uptake into T-lymphocytes at 37°C after 7 days in culture. ●, 37°C control; ○, 4°C control; □, 1.0 $\mu$M 555; ■, 5.0 $\mu$M 555; △, 0.5 $\mu$M DP; ▲, 1.0 $\mu$M DP. Points are means of duplicate cultures.

*Cytotoxicity of DP and 555*

The effect of the nucleoside transport inhibitors on PBL growth at high and low plating densities was examined. High density studies utilised PBLs plated into microwells at $10^4$ cells per well in NM and harvested at intervals over a 12-day period. Control PBL growth at this cell density plateaued after 7 days and was unaffected by 555 concentrations $\leq 0.03$ $\mu$M, but concentrations $\geq 0.12$ $\mu$M reduced growth markedly after the first two days of culture (Figure 2). By day 12 viable cell numbers were lower than the initial plating density. On the other hand DP (1.0–5.0 $\mu$M) had no effect on PBL growth at high plating densities over a similar culture period.

For low density studies, PBLs were plated in NM at limiting dilution in the presence or absence of various concentrations of either inhibitor. PBL cloning efficiencies were not affected by 0.1 $\mu$M 555, but 0.25 and 0.5 $\mu$M 555 reduced cloning to 82% and 75% of control respectively. On the other hand 1.0–5.0 $\mu$M DP decreased the cloning efficiencies to 80% of control values.

*MTX and DP or 555*

MTX cytotoxicity in the presence of either transport inhibitor was also studied. $10^4$ target cells were plated per well in NM in the presence or absence of 100 $\mu$M MTX. Under these conditions MTX-induced cell death plateaued after 5 days of culture (Figure 3). MTX cytotoxicity was unaffected by 555 concentrations $\leq 0.03$ $\mu$M. However, cytotoxicity was increased by 555 $\geq 0.13$ $\mu$M (Figure 3a). DP 1.0–5.0 $\mu$M also potentiated MTX cytotoxicity (Figure 3b).

The effects of 555 and DP on nucleoside salvage of MTX cytotoxicity was investigated. PBL microwell cultures were set up in duplicate at $10^2$ cells per well in DM and harvested over a 12-day period for viable cell counts. Under these conditions 100 $\mu$M MTX was completely cytotoxic to PBLs. However, cultures supplemented with TdR (1.0 $\mu$M) and Hx

**Figure 2** PBL growth at high density in NM in microwells with $10^4$ feeder cells per well in the presence (——) or absence (- - -) of 555. ○, control; ●, 0.003 $\mu$M; ▲, 0.03 $\mu$M; △, 0.125 $\mu$M; ■, 0.25 $\mu$M; □, 0.5 $\mu$M 555. Points are means of duplicate well cultures. Bars are standard errors of means from 3–5 experiments.

**Figure 3** (**a**) MTX cytotoxicity in PBLs grown at high density in NM in microwells with $10^4$ feeder cells per well in the presence (——) or absence (– – –) of 555. ○, control; △, 100 μM MTX; ▲, 0.03 μM 555 + 100 μM MTX; ●, 0.125 μM + 100 μM MTX; ■, 0.25 μM 555 + 100 μM MTX; □, 0.5 μM 555 + 100 μM MTX. Points are means of duplicate cultures. Bars are standard errors of means from 3–5 experiments. (**b**) MTX cytotoxicity in PBLs grown at high density in NM in microwells with $10^4$ feeder cells per well in the presence (——) or absence (– – –) of DP. ○, control; △, 100 μM MTX; ■, 1.0 μM DP + 100 μM MTX; ▲, 2.5 μM DP + 100 μM MTX; ●, 5.0 μM DP + 100 μM MTX. Bars are standard errors of means from 4–5 experiments.

**Figure 4** The effect of 555 and DP on PBLs grown at low density in DM in microwells with $10^4$ feeder cells per well in the presence (——) or absence (– – –) of 1.0 μM TdR, 1.0 μM Hx and 100 μM MTX (MTH). – – ○ – –, control; —●—, MTH; – – △ – –, 1.0 μM DP; – – ▲ – –, 5.0 μM DP; – – ■ – –, 0.1 μM 555; —■—, 0.1 μM 555 + MTH. No viable cells were detected in wells containing any of the following: – – ● – –, 100 μM MTX; – – □ – –, 1.0 μM 555; —□—, 1.0 μM 555 + MTH; —△—, 1.0 μM DP + MTH; —▲—, 5.0 μM DP + MTH. Points are means of two experiments. Bars are standard errors of means from 3–4 experiments.

(1.0 μM) maintained a substantial level of growth in the presence of 100 μM MTX (MTH) (Figure 4). The addition of 0.1 μM 555 reduced this level of growth while 1.0 μM 555 or 1.0–5.0 μM DP completely inhibited it. However, in the absence of TdR, Hx and MTX (MTH), 0.1 μM 555 inhibited normal growth and 1.0 μM 555 was lethal. DP, 1.0 μM, had no effect on growth in DM at these cell densities in the absence of MTX while 5.0 μM DP reduced it slightly.

### Discussion

The studies reported here demonstrate that both DP and 555 potentiate MTX cytotoxicity in PBLs *in vitro*. DP, 1.0 μM, abolished $^3$H-TdR uptake, potentiated MTX cytotoxicity and reversed TdR modulation of MTX cytotoxicity in stimulated PBLs (Figures 1, 3b and 4, respectively). DP alone, up to 5.0 μM, did not affect PBL growth at high cell density and only slightly decreased growth when cells were plated at lower densities (Figure 4).

6-(2-hydroxy-5-nitrobenzyl)thioguanosine (555) potentiated MTX cytotoxicity (Figure 3c) but was itself cytotoxic at concentrations which inhibited TdR uptake. Under these culture conditions 555 was a less potent inhibitor of TdR uptake in PBLs than DP, with ⩾5.0 μM 555 being required.

555, >0.13 μM, substantially decreased growth in cells plated at high (Figure 2) or low density, and particularly at low density in medium with dialysed serum (Figure 4). As the concentration of 555 which inhibited TdR uptake was at least 10 times greater than that which affected PBL growth, we conclude that its cytotoxicity is probably unrelated to TdR transport inhibition.

In cell culture the levels of exogenous purines and pyrimidines are greatly influenced by the serum used (Sobrero & Bertino, 1986; Piper *et al.*, 1983). In addition nucleosides may be released into the culture medium from damaged cells (Hughes *et al.*, 1988). Figure 4 illustrates that DP reversed the TdR modulation of MTX cytotoxicity and is therefore a practical alternative to utilising dialysed serum when large numbers of cells are being screened, as in chemosensitivity testing. DP and other nucleoside transport inhibitors may also help define the cytotoxic mechanisms of antimetabolites such as MTX. DP is to be preferred to 555 since 555 is growth inhibitory by itself.

Potentiation of antimetabolite cytotoxicity by DP or other nucleoside transport inhibitors has relevance *in vivo*. TdR and Hx are present in plasma samples from normal subjects and cancer patients (Howell *et al.*, 1981). Regional variations in nucleoside and base concentrations have been reported *in vivo*, e.g. substantially greater levels of hypoxanthine-xanthine being detected in bone marrow than in peripheral plasma (Tattersall *et al.*, 1983). Anoxic cell death in avascular areas of a tumour may increase nucleoside and base levels locally. The relative activities of the rate-limiting enzymes of the pyrimidine *de novo* and salvage pathways are increased in neoplastic liver cells compared with normal cells (Weber, 1983). Moreover, Weber (1983) has suggested that agents such as DP, in combination with the appropriate antimetabolite, may be effective for drug refractory tumours like colon cancer, because of their very active salvage pathways. Oral DP and low dose oral MTX showed some antitimour activity in lung and breast cancer patients in a phase I study (Subar *et al.*, 1986), but the combination was ineffective in a phase II trial in patients with advanced colorectal carcinoma (Wadler *et al.*, 1987). The authors postulated that this was due to incomplete inhibition of nucleoside salvage by oral DP and advocated further studies using i.v. infusion of DP. The possible reduction in antimetabolite cytotoxicity due to metabolite salvage is a compelling reason for further investigation of the potential role for nucleoside transport inhibitors, such as DP, in modulating nucleic acid antimetabolite drug action *in vivo*.

J.M. Hughes was supported by a NH and MRC Biomedical Scholarship for part of this study. The authors thank Dr Anna deFazio and Mr Peter Slowiaczek for their help and advice, and Miss Judy Hood for typing this manuscript.

### References

BASTIDA, E., DEL PRADO, J., ALMIRALL, R., JAMIESON, G.A. & ORDINAS, A. (1985). Inhibitory effects of dipyridamole on growth, nucleoside incorporation, and platelet-activating capability in the U87MG and SKNMC human tumor cell lines. *Cancer Res.*, **45**, 4048.

BRAJESWAR, P., CHEN, M.F. & PATERSON, A.R.P. (1975). Inhibitors of nucleoside transport. A structure-activity study using human erythrocytes. *J. Med. Chem.*, **18**, 968.

FISCHER, P.H., PAMUKCU, R., BITTNER, G. & WILLSON, J.K. (1984). Enhancement of the sensitivity of human colon cancer cells to growth inhibition by acivicin achieved through inhibition of nucleic acid precursor salvage by dipyridamole. *Cancer Res.*, **45**, 3355.

GREM, J.L. & FISCHER, P.H. (1986). Alteration of fluorouracil metabolism in human colon cancer cells by dipyridamole with a selective increase in fluorodeoxyuridine monophosphate levels. *Cancer Res.*, **46**, 6191.

HOWELL, S.B., MANSFIELD, S.J. & TAETLE, R. (1981). Thymidine and hypoxanthine requirements of normal and malignant human cells for protection against methotrexate cytotoxicity. *Cancer Res.*, **41**, 945.

HUGHES, J.M., DEFAZIO, A. & TATTERSALL, M.H.N. (1988). Modulation of antifolate cytotoxicity by metabolites from dying cells in a lymphocyte clonal assay. *Br. J. Cancer.*, **57**, 459.

MIZEL, S.B. & WILSON, L. (1972). Nucleoside transport in mammalian cells. Inhibition by colchicine. *Biochemistry* **11**, 2573.

MUGGIA, F.M., SLOWIACZEK, P. & TATTERSALL, M.H.N. (1987). Characterization of conditions in which dipyridamole enhances methotrexate toxicity in L1210 cells. *Anticancer Res.*, **7**, 161.

PIPER, A.A., NOTT, S.E., MACKINNON, W.B. & TATTERSALL, M.H.N. (1983). Critical modulation by thymidine and hypoxanthine of sequential methotrexate-5-fluorouracil synergism in murine L1210 cells. *Cancer Res.*, **43**, 5701.

PLAGEMANN, P.G.W. & ESTERSEN, R.D. (1972). Cytochalasin B. VI. Competitive inhibition of nucleoside transport by cultured Novikoff rat hepatoma cells. *J. Cell. Biol.*, **55**, 179.

PLAGEMANN, P.G.W. & WOHLHUETER, R.M. (1984). Nucleoside transport in cultured mammalian cells. Multiple forms with different sensitivity to inhibition by nitrobenzylthioinosine or hypoxanthine. *Biochim. Biophys. Acta*, **773**, 39.

PLAGEMANN, P.G.W. & WOHLHUETER, R.W. (1985). Effects of nucleoside transport inhibitors on the salvage and toxicity of adenosine and deoxyadenosine in L1210 and P388 mouse leukemia cells. *Cancer Res.*, **45**, 6418.

SOBRERO, A.F., MOIR, R.D., BERTINO, J.R. & HANDSCHUMACHER, R.E. (1985). Defective facilitated diffusion of nucleosides, a primary mechanism of resistance to 5-fluoro-2′-deoxyuridine in the HCT-8 human carcinoma line. *Cancer Res.*, **45**, 3155.

SOBRERO, A.F. & BERTINO, J.R. (1986). Endogenous thymidine and hypoxanthine are a source of error in evaluating methotrexate cytotoxicity by clonogenic assays using undialyzed fetal bovine serum. *Int. J. Cell Cloning*, **4**, 51.

SUBAR, M., MUGGIA, F., GREEN, M.D. & FISCHER, P. (1986). Phase I study of daily oral methotrexate with concurrent dipyridamole (DP) for inhibition of salvage pathway 'rescue'. *Proc. ASCO*, **5**, 42.

TASWELL, C. (1981). Limiting dilution assays for the determination of immunocompetent cell frequencies. I. Data analysis. *J. Immunol.*, **126**, 1614.

TATTERSALL, M.H.N., SLOWIACZEK, P. & DEFAZIO, A. (1983). Regional variation in human extracellular purine levels. *J. Lab. Clin. Med.*, **102**, 411.

TAYLOR, I.W. & TATTERSALL, M.H.N. (1981). Methotrexate cytotoxicity in cultured human leukaemic cells studied by flow cytometry, *Cancer Res.*, **41**, 1549.

VAN MOUWERIK, T.J., PANGALLO, C.A., WILLSON, J.K.V. & FISCHER, P.H. (1987). Augmentation of methotrexate cytotoxicity in human colon cancer cells achieved through inhibition of thymidine salvaged by dipyridamole. *Biochem. Pharmacol.*, **36**, 809.

WADLER, S., SUBAR, M., GREEN, M.D., WIERNIK, P.H. & MUGGIA, F.M. (1987). Phase II trial of oral methotrexate and dipyridamole in colorectal carcinoma. *Cancer Treat. Rep.*, **71**, 821.

WEBER, G. (1983). Biochemical strategy of cancer cells and the design of chemotherapy: G.H.A. Clowes memorial lecture. *Cancer Res.*, **43**, 3466.